# Bilateral sudden sensorineural hearing loss with Sweet syndrome: A case report

**DOI:** 10.1097/MD.0000000000022127

**Published:** 2020-09-04

**Authors:** Yeon Seok You, Sang Woo Park, Seok Kweon Yun, Eun Jung Lee

**Affiliations:** a Department of Otorhinolaryngology-Head and Neck Surgery; b Department of Dermatology, Chonbuk National University, School of Medicine; c Research Institute of Clinical Medicine of Chonbuk National University, Biomedical Research Institute of Chonbuk National University Hospital, Jeon-ju, Republic of Korea.

**Keywords:** bilateral, hearing loss, sensorineural, Sweet syndrome

## Abstract

**Introduction:**

Sweet syndrome (SS) is an idiopathic autoimmune disease which has been associated with various extracutaneous manifestations. Otologic symptoms secondary to SS are characterized by bilateral, progressive, sensorineural hearing loss, which requires auditory rehabilitation with, for example, cochlear implantation.

**Patient concerns:**

A 43-year-old woman complaining of bilateral sudden hearing loss visited the Emergency Department of our University. Abrupt onset of fever peaking up to 40°C and vomiting accompanied the hearing loss and other associated symptoms were: tinnitus that sounded like a machine humming, mild dizziness, a painful rash (on the right upper eyelid, chest, back, forearms, and lower extremities), arthralgia in both the hip and knee joints, and vision loss in the right eye. The patient had no history of autoimmune diseases or surgery.

**Diagnosis:**

Pure tone audiometry and biopsy on the skin lesion were performed. SS with bilateral sudden sensorineural hearing loss was confirmed.

**Interventions:**

The patient was treated with intravenous prednisolone and topical steroids.

**Outcomes:**

After a week of treatment, skin lesions had improved. And 3 months after treatment, the hearing test showed full recovery.

**Conclusion:**

This case emphasizes the point that early diagnosis and timely treatment are essential for hearing recovery in patients with SS who have otologic symptoms.

## 1 Introduction

Sweet syndrome (SS), also known as acute febrile neutrophilic dermatosis and which is often associated with immunological disease, is characterized by clinical manifestations comprising pyrexia, painful erythematous nodules or plaques, and a dense neutrophilic infiltrate with the absence of leukocytoclastic vasculitis.^[1]^ At the present time, the pathogenesis of SS is unclear, but it is reported to be related to systemic causes including cancer, infections, inflammatory bowel disease, medications, or pregnancy.^[1]^ The extracutaneous manifestations of SS can affect various organs including the central nervous system, eyes, kidneys, heart, and lungs.^[1,2]^ As a result of associated autoimmune disorders, SS can affect the inner ear and cause bilateral hearing loss. To date, there have only been 3 reports in the English literature of bilateral progressive hearing loss associated with SS.^[3–5]^ In these reports, SS is associated with secondary bilateral, progressive, sensorineural hearing loss, which required auditory rehabilitation with, for example, hearing aids or cochlear implants. To the best of our knowledge, there have been no reports of SS occurring simultaneously with bilateral sudden sensorineural hearing loss (SSNHL).

In the present study, we report a patient with bilateral SSNHL that occurred simultaneously with the diagnosis of SS. The patient immediately received systemic steroids and an intra-tympanic steroid injection, and after 3 months, showed complete recovery of hearing.

This study was approved by the institutional review board of Chonbuk National University Hospital Committee, Korea: CUH 2019-03-029. Informed written consent was obtained from the patient for publication of this case report and accompanying images.

## 2 Case report

A 43-year-old woman visited the Emergency Department (ED) of our University with bilateral sudden hearing impairment. The hearing loss had occurred suddenly with fever and vomiting a day before the visit to the ED and associated symptoms were: tinnitus that seemed like a machine humming, mild dizziness, fever peaking at up to 40°C, a painful rash (on the right upper eyelid, chest, back, forearms, and lower extremities), arthralgia in both the hip and knee joints, and vision loss in the right eye. The patient had no history of autoimmune diseases or surgery.

**Figure 1:**
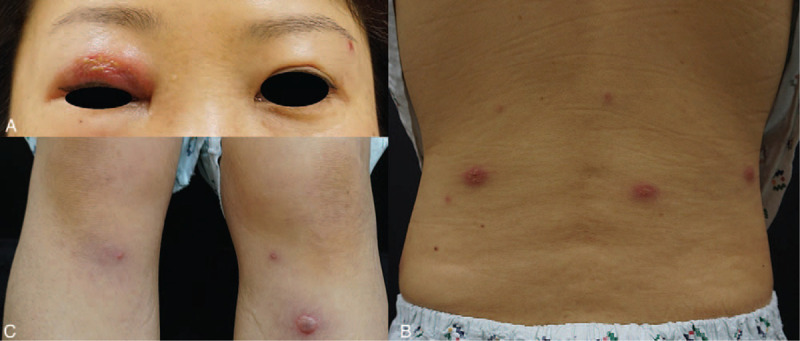
(A) The 42-year-old woman in this study developed a tender erythematous swelling on the right upper eyelid. (B, C) Multiple swelling erythematous papulonodules on her back and lower legs. Some of the lesions gave rise to vesicles.

Upon physical examination, both tympanic membranes were normal, and examination with a tuning fork showed left lateralization for the Weber test, and both ears were positive for the Rinne test. A rash could be seen all over her body, and her eyesight was measured to be 0.2 in the right eye, and 0.9 in the left eye (Fig. 1).

**Figure 2:**
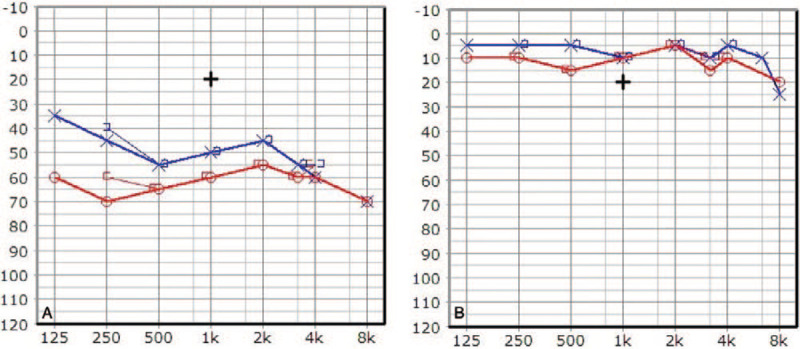
Pure tone audiogram (blue line indicates the left hearing threshold and red line indicates the right hearing threshold). (A) Bilateral moderate to severe hearing loss was seen on the pure tone audiogram before treatment. (B) After 3 months, the hearing test showed full recovery: 11 dB in the right ear, and 8 dB in the left ear.

**Figure 3:**
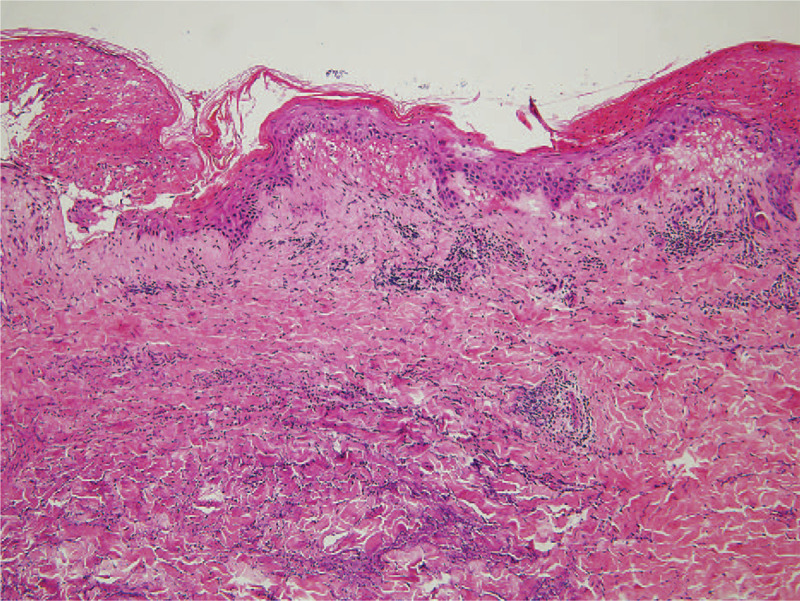
An inflammatory cell infiltrate composed of neutrophils is seen assuming a band-like distribution on the upper dermis. There is edema of the papillary dermis (H&E, ×100).

Laboratory test results were: high-sensitivity C-reactive protein (hsCRP) 160.12 mg/L, erythrocyte sedimentation rate (ESR) 79 mm/hr, and Seg neutrophil 84.10%. Pure tone audiometry (PTA) was performed on the second day after hearing loss had occurred (Fig. 2A), and showed bilateral sensorineural hearing loss. For a diagnosis of SS, clinical symptoms must satisfy both of 2 major criteria, and more than 2 of 4 minor criteria (Table 1). Our patient had abrupt onset of painful erythematous plaques which met the major criteria, with pyrexia (>38°C) and abnormal laboratory values (3/4; erythrocyte sedimentation rate >20 mm/h, positive C-reactive protein, >8000 leukocytes, and >70% neutrophils) that satisfied the minor criteria. For a definitive diagnosis, we performed a skin biopsy with samples from the skin on her back; the results were consistent with acute febrile neutrophilic dermatosis (Fig. 3). Intravenous prednisolone was given, and topical steroids were applied to the skin lesions.

**Table 1:**
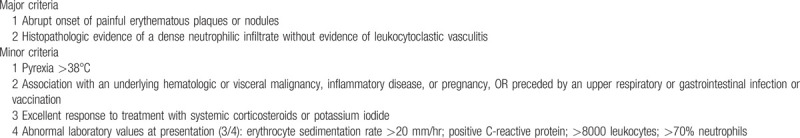
Diagnostic criteria for Sweet syndrome.

There were no remarkable findings from brain magnetic resonance imaging and magnetic resonance angiography, but right orbital cellulitis, as shown by the diffuse soft tissue swelling and enhancement in the right upper and lower eyelids, was found on facial computed tomography (CT). After a week of treatment, there was no change in vision but skin lesions had improved. Follow-up on the hearing test showed an improvement of up to 20 dB in both ears. The patient was discharged after a week of treatment, with follow-up at the outpatient clinic after 1 month. Vision examination performed at the outpatient clinic showed a vast improvement to 1.2 in the right eye, and 1.2 in the left eye. After 3 months, the hearing test showed full recovery: 11 dB in the right ear, and 8 dB in the left ear (Fig. 2B).

## 3 Discussion

SS was originally described by Dr Robert Douglas Sweet in 1964.^[6]^ The etiology is thought to be multifactorial and further research is needed. Currently, systemic corticosteroids are considered the criterion standard treatment for this disease^[1]^ and immediate clinical improvement with steroid administration, and immunosuppressants, suggests that the syndrome may be related to an immunological pathogenesis.^[1,4,6]^ In addition, clinical evidence and laboratory results in recent studies suggest that cytokine effects also have an etiological role.^[2]^ The occurrence of bilateral sudden sensorineural hearing loss is rare compared to unilateral sensorineural hearing loss. In addition, bilateral sudden sensorineural hearing loss is more likely to be due to the patient's general condition rather than to idiopathic causes, with more severe hearing loss and poorer prognosis than with unilateral sudden sensorineural hearing loss.^[7]^ Considering the immunological pathogenesis of SS, bilateral sudden sensorineural hearing loss in SS may be due to systemic conditions which eventually affect the inner ear.

Up to now, research conducted in other countries has reported three cases of progressive bilateral sensorineural hearing loss with SS. All patients from those three cases had a history of chronic SS, and despite many attempts including systemic steroid therapy, multiple steroid sparing agents and cochlear implants, the progress of bilateral sensorineural hearing loss could not be stopped.^[3–5]^

The case presented here is the first which has shown full recovery of hearing after bilateral sensorineural hearing loss, and which was enabled by early and immediate diagnosis, and appropriate steroid administration.

It must be noted that patients in the 3 previous cases showed no signs of sensorineural hearing loss at the time SS was initially diagnosed. It can be hypothesized that autoimmunity had not yet affected the inner ear in the initial phase of SS, and the patients’ condition at some point during the progress of the disease triggered the gradual damage. In comparison, our patient showed bilateral SSNHL from the time of initial diagnosis, with full recovery of hearing after immediate steroid injection.

In this case, the patient showed an immediate response to systemic steroids, and symptoms—including hearing loss—were completely reversed. This supports the existing evidence that steroid therapy is a criterion standard for SS and that it is thus associated with an immunological pathogenesis.

As most cases up to now did not receive treatment at an early stage of the disease, we suggest that the failure to improve bilateral sensorineural loss in previous cases may be due to delayed treatment. Thus, time-appropriate treatment is crucial for hearing recovery, and patients who are suspected of having SS should undergo a thorough history and physical examination for early diagnosis. Most importantly, when clinical evidence indicates SS, clinicians should not hesitate to perform a hearing test as soon as possible, regardless of symptoms. In addition, patients with SS should be fully warned about the possibility of bilateral hearing loss, and advised to adhere to a periodic examination by an otolaryngologist, even when symptoms are not present.

## Author contributions

**Conceptualization:** Yeon Seok You, Eun Jung Lee.

**Data curation:** Yeon Seok You.

**Investigation:** Yeon Seok You.

**Methodology:** Yeon Seok You, Eun Jung Lee.

**Project administration:** Yeon Seok You, Eun Jung Lee.

**Resources:** Yeon Seok You, Sang Woo Park, Seok Kweon Yun.

**Supervision:** Yeon Seok You, Eun Jung Lee.

**Validation:** Yeon Seok You, Eun Jung Lee.

**Visualization:** Yeon Seok You, Sang Woo Park, Seok Kweon Yun.

**Writing – original draft:** Yeon Seok You.

**Writing – review & editing:** Yeon Seok You, Eun Jung Lee.
